# Activation of BMP-Smad1/5/8 Signaling Promotes Survival of Retinal Ganglion Cells after Damage *In Vivo*

**DOI:** 10.1371/journal.pone.0038690

**Published:** 2012-06-06

**Authors:** Yumi Ueki, Thomas A. Reh

**Affiliations:** Department of Biological Structure, University of Washington, Seattle, Washington, United States of America; Morehouse School of Medicine, United States of America

## Abstract

While the essential role of bone morphogenetic protein (BMP) signaling in nervous system development is well established, its function in the adult CNS is poorly understood. We investigated the role of BMP signaling in the adult mouse retina following damage *in vivo*. Intravitreal injection of N-Methyl-D-aspartic acid (NMDA) induced extensive retinal ganglion cell death by 2 days. During this period, BMP2, -4 and -7 were upregulated, leading to phosphorylation of the downstream effector, Smad1/5/8 in the inner retina, including in retinal ganglion cells. Expression of Inhibitor of differentiation 1 (Id1; a known BMP-Smad1/5/8 target) was also upregulated in the retina. This activation of BMP-Smad1/5/8 signaling was also observed following light damage, suggesting that it is a general response to retinal injuries. Co-injection of BMP inhibitors with NMDA effectively blocked the damage-induced BMP-Smad1/5/8 activation and led to further cell death of retinal ganglion cells, when compared with NMDA injection alone. Moreover, treatment of the retina with exogenous BMP4 along with NMDA damage led to a significant rescue of retinal ganglion cells. These data demonstrate that BMP-Smad1/5/8 signaling is neuroprotective for retinal ganglion cells after damage, and suggest that stimulation of this pathway can serve as a potential target for neuroprotective therapies in retinal ganglion cell diseases, such as glaucoma.

## Introduction

Injury to the central nervous system (CNS) causes the release of endogenous neuroprotective factors. In the retina, photoreceptors near the site of mechanical injury are protected against light-induced death in rat retinas [1] by the up-regulation of fibroblast growth factor 2 (FGF2) and ciliary neurotrophic factor (CNTF) [2], [3]. Genetic models of retinal degeneration show increased FGF2 and leukemia inhibitory factor (LIF) expression [4], [5]. Although the endogenous increase in expression of these factors provides a degree of neuroprotection, treatment of the retina with exogenous neurotrophic factors can further potentiate neuronal survival in retinal damage. Intravitreal delivery of LIF, CNTF, brain-derived neurotrophic factor (BDNF), or FGF2 promotes survival of photoreceptors after light damage or inherited retinal degeneration, and protects retinal ganglion cells after ischemic injury [6], [7], [8], [9], [10].

Although the neurotrophic properties of FGF2, CNTF, LIF, and BDNF have been well studied, bone morphogenetic proteins (BMPs) have received less attention. BMPs constitute a large family of related proteins within the larger transforming growth factor beta (TGF-beta) superfamily [11]. Canonical BMP signaling is activated by the binding of BMPs to heterodimeric complexes of type I and type II cell surface receptors (BMPRI and BMPRII), which in turn leads to phosphorylation of Smad1/5/8. When phosphorylated, Smad1/5/8 forms a complex with Smad4, which translocates to the nucleus to regulate target gene transcription.

BMP has important functions in early nervous system development [12]. In addition to these developmental roles, several studies have shown that BMP signaling increases after both acute damage and neurodegenerative disorders. For example, BMP4 and −7 rapidly increase after spinal cord injury in rat, with phosphorylation of Smad1/5/8 at the injury site [13], [14]. Increased BMP6 levels have also been detected in brains of amyloid precursor protein transgenic mice and Alzheimer's disease patients [15].

Although BMPs are known to play essential roles in retinal development [16], [17], [18], [19], [20], [21], [22], it is not known whether the changes in BMP signaling that occur in other regions of the CNS also take place in damaged retinas. Therefore, we investigated potential roles for BMP signaling after injury in mouse retina. We find that retinal injury causes an up-regulation in BMP expression, and Smad1/5/8 phosphorylation in the inner retina. This activation of BMP-Smad1/5/8 signaling is neuroprotective for retinal ganglion cells; inhibition of BMP signaling after NMDA treatment significantly reduces the number of surviving retinal ganglion cells. Moreover, treatment with exogenous BMP4 promotes survival of retinal ganglion cells after NMDA damage. Thus, activation of BMP-Smad1/5/8 may provide an additional neuroprotective target for retinal degenerations.

## Materials and Methods

### Mice

Mice were housed in the University of Washington Department of Comparative Medicine and the University of Washington Institutional Animal Care and Use Committee (UW-IACUC) approved the animal housing and the experimental protocols used in this study (Protocol # 2448-08). Mice used for NMDA damage experiments were 6–8 week old C57BL/6. 6–8 week old Swiss Webster (albino) mice were used for light damage experiments. Hes5-GFP transgenic mice [23], which express GFP in Müller glia in the adult retina [24], were used for some experiments.

### NMDA damage and intravitreal injection

Mice were deeply anesthetized with a single intraperitoneal injection of ketamine (130 mg/kg) and xylazine (8.8 mg/kg). Two different doses of NMDA, either 10 or 100 mM along with the other indicated factors were delivered by a single intravitreal injection. The estimated concentration of NMDA in the eye after the injection was 2.8 mM for the low dose and 28 mM for the high dose. Intravitreal injection was performed using a 32 gauge needle (Hamilton, Reno, NV) through the temporal limbus of the eye. Each eye received 2 µL of indicated factors or PBS (vehicle control). Any eye showing signs of damage due to intravitreal injection, such as inflammation and morphological disruption was excluded from analyses. Factors injected were: 25 ng/µL mouse recombinant BMP4 (R&D systems), 1 mM dorsomorphin (Tocris), 25 ng/µL noggin (R&D systems), and/or 0.5 mM LDN-193189 (Stemgent).

### Light damage

Unanesthetized albino mice were exposed to diffuse, cool, white fluorescent light coming from the top of the cage. Food and water were provided *ad libitum* but were placed in the cage to avoid blocking light exposure. The pupils were not dilated. Average luminance was measured on the cage floor using a light meter, and was approximately 10,000 lux. Mice were exposed to the light for 8 hrs (8am–4pm), and returned under normal lighting (12 hr on/12 hr off cyclic light) for recovery before analysis.

### Immunohistochemistry

Eyes were enucleated and fixed in 2% paraformaldehyde in PBS for 45 min at room temperature immediately after animals were euthanized with CO_2_. The cornea and lens were removed during the fixation period. For cross sections, eye cups were cryoprotected in increasing concentrations of sucrose (10–30%) in PBS at room temperature, followed by overnight incubation at 4°C in PBS containing 30% sucrose. Eyes were embedded in Tissue-Tek OCT Compound (Sakura Fintek, Torrance, CA), rapidly frozen on dry ice, and then sectioned on a cryostat at 12–14 µm. Slides were dipped in −20°C methanol briefly, dried at room temperature, and stored at −20°C until use. For retinal flatmounts, retinas were isolated after the fixation period, and washed once with PBS before performing immunohistochemistry.

For immunohistochemistry, slides or retinas were washed once with PBS containing 1% Triton X-100. Nonspecific binding was blocked by incubating sections or retinas with 10% horse serum in PBS for at least 1 hr at room temperature, and primary antibody was applied overnight at 4°C. Primary antibodies used were: rabbit anti-GFAP (Dako), goat anti-Brn3 (Santa Cruz Biotechnology), mouse anti-HuC/D (invitrogen), rabbit anti-pSmad1/5/8, rabbit anti-pSmad2/3 (Cell Signaling), and rabbit anti-Id1 (BioCheck). Specimens were washed, and incubated with Goat or donkey anti-rabbit Alexa Fluor 568 (Invitrogen), goat anti-mouse 488 (Invitrogen), or donkey anti-goat 568 (Invitrogen) antibody for 2 hrs at room temperature. Nuclei were counterstained with DAPI (Sigma) and sections or retinal flatmounts were coverslipped with PBS containing 50% glycerol. Imaging was performed using an Olympus FluoView confocal laser scanning microscope. To ensure quantitative image quality, laser power, pinhole settings, PMT settings, and intensity thresholds were kept constant for a given antibody.

### Retinal ganglion cell counts

One 3-µm, single slice confocal image was taken in a 0.4 mm^2^ random field for each retinal flatmount (approximately one eighth of the whole retinal area), and the number of Brn3+ cells in each image was counted manually. The number of Brn3+ cells in one mm^2^ of the retina was calculated and plotted. The number of animals used for each treatment group is indicated in each figure. Statistics were performed usinga Student's t-test, and error bars indicate SEM.

### Real-time qPCR

The right eye of each mouse was injected with NMDA, and the left eye was left untreated as a control. Each RNA sample was from a single retina. Total RNA was isolated from each retina using Trizol (Invitrogen). RNA was treated with RQ1 DNase (Promega), and then purified with RNeasy kit (Qiagen). cDNA synthesis was performed using iScript cDNA synthesis kit (Biorad), and real-time qPCR was performed using SsoFast EvaGreen Supermix (Biorad). Gapdh was used as a normalization control. Fold change in expression was calculated for each pair of samples (NT vs NMDA), and plotted. Statistics were performed using a paired t-test, and error bars represents SEM. Primer sequences used were as follows: *Bmp2* F 5′-GGGACCCGCTGTCTTCTAGT-3′, *Bmp2* R 5′-TCAACTCAAATTCGCTGAGGAC-3′, *Bmp4* F 5′-GACTTCGAGGCGACACTTCTA-3′, *Bmp4* R 5′-GCCGGTAAAGATCCCTCATGTAA-3′, *Bmp5* F 5′-TTACTTAGGGGTATTGTGGGCT-3′, *Bmp5* R 5′- CCGTCTCTCATGGTTCCGTAG-3′, *Bmp7* F 5′-ACGGACAGGGCTTCTCCTAC-3′, *Bmp7* R 5′-ATGGTGGTATCGAGGGTGGAA-3′, *Gapdh* F 5′-GGCATTGCTCTCAATGACAA-3′, and *Gapdh* R 5′-CTTGCTCAGTGTCCTTGCTG-3′.

### Western blot

Retinas were harvested immediately after animals were euthanized by CO_2_ asphyxiation, and homogenized in lysis buffer [50 mM Tris-HCl (pH 7.5), 150 mM NaCl, 5 mM EDTA, 1% (v/v) NP-40, 5% (v/v) glycerol, phosphatase inhibitor cocktail (Roche), and protease inhibitor cocktail (Sigma)]. One retina was used per each sample. Equal amounts of total protein were electrophoresed on 4–20% gradient SDS-polyacrylamide gels (BioRad), and transferred to PVDF membranes (BioRad). The membranes were blocked with 5% bovine serum albumin (BSA) for 1 hr at room temperature, and then incubated overnight at 4°C with indicated antibodies. Antibodies used were: rabbit anti-Id1 (Biocheck) and mouse anti-beta actin (Abcam). Membranes were then incubated for 1 hr at room temperature with HRP-conjugated anti-rabbit or anti-mouse secondary antibodies (BioRad). Signals were exposed to X-ray films using SuperSignal West Dura Extended Duration Substrate (Thermo Scientific). Densitometry analyses were performed using ImageJ. Id1 expression was normalized to beta-actin, and plotted. Statistics were performed using t-test, and error bars indicate SEM.

## Results

### Intravitreal injection of NMDA induces retinal ganglion cell death

**Figure 1:**
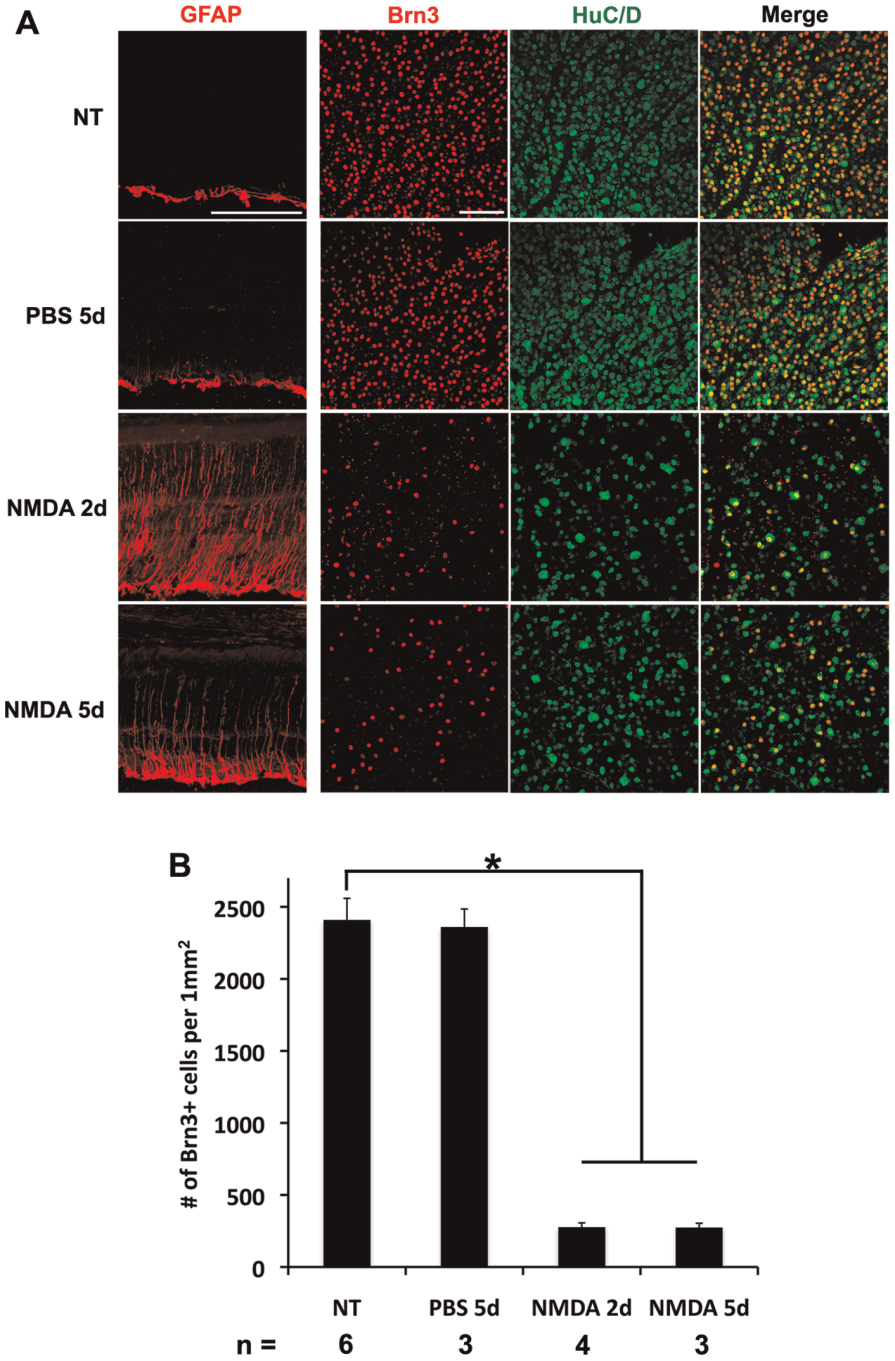
Intravitreal injection of NMDA results in death of retinal ganglion cells and amacrine cells by 2 days. **A.** Images of retinal cross sections (left column) and flatmounts (right 3 columns) are shown. Neurotoxic damage induced by 100 mM NMDA injection caused a reduction in Brn3+ (red) retinal ganglion cells and HuC/D+ amacrine (green) cells compared to untreated (NT) or vehicle injected (PBS) retinas by 2 days after injection. GFAP, a marker for retinal damage, was also upregulated at 2 days. Scale bars: 100 µm. **B.** Brn3+ cells were counted in random flatmount fields. At 2 days after NMDA injection, there was a significant reduction in Brn3+ cells (276.5±29.6 Brn3+ cells/mm^2^) compared to NT retinas (2409.5±149.9 Brn3+ cells/mm^2^). No further reduction in Brn3+ cells was observed at 5d after NMDA injection (273.7±29.6 Brn3+ cells/mm^2^). Therefore, we collected eyes 2 days after injection for the rest of the study. *p<0.005 with t-test.

Previous studies have shown that intravitreal injections of NMDA cause rapid death of retinal ganglion cells [25], [26]. To confirm these earlier studies, we made injections of high dose of NMDA (100 mM) into the vitreal chamber of adult mice, and analyzed the retinas 2–5 days later in sections and flatmounts. We found that by 2 days after the NMDA treatment, there was already a substantial reduction in the number of Brn3+ retinal ganglion cells (276.5±29.6 Brn3+ cells/mm^2^), when compared with uninjected (2409.5±149.9 Brn3+ cells/mm^2^) or PBS injected control retinas (2277.6±59.0 Brn3+ cells/mm^2^) (Figure 1A–B). There was a similar reduction in the number of another marker of ganglion and amacrine cells, HuC/D [27], in the NMDA treated retinas. Both Brn3 and HuC/D showed similar declines after 5 days (273.7±29.6 Brn3+ cells/mm^2^) (Figure 1A–B). The reduction in the number of ganglion cells was paralleled by an increase in the expression of GFAP, a marker for retinal damage, in the Müller glial cells (Figure 1A). These results indicate that intravitreal injections of 100 mM NMDA induce rapid death of retinal ganglion cells in adult mice.

### Retinal damage activates BMP-Smad1/5/8 signaling in the inner retina, including Müller glia and retinal ganglion cells

**Figure 2:**
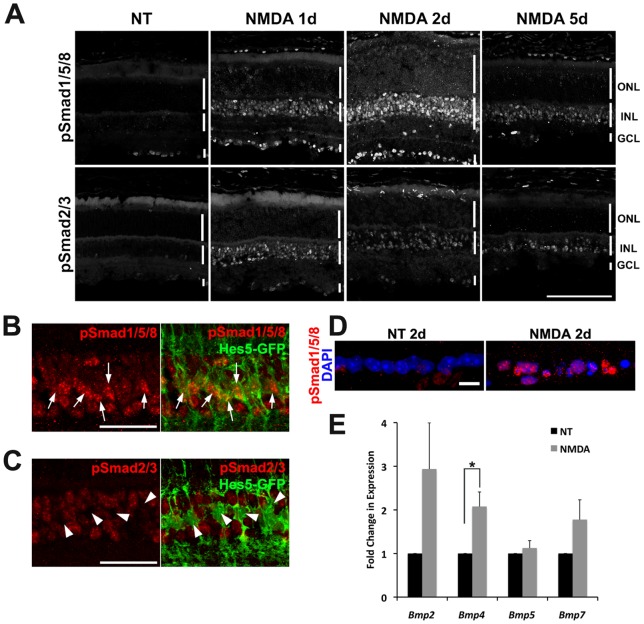
Retinal damage induces Smad phosphorylation in retinal ganglion cells and inner retinal cells, and increases BMP mRNA expression in the retina. A. Injection of 100 mM NMDA induced Smad1/5/8 activation (pSmad1/5/8) in the retinal ganglion cells and inner retinal cells. The peak of activation was observed at 2 days. Smad2/3 was also activated (pSmad2/3) in the inner retina to a lesser extent. Scale bar: 100 µm. ONL, outer nuclear layer; INL, inner nuclear layer; GCL, ganclion cell layer. **B–C.** While strong Smad1/5/8 activation was induced in Hes5-GFP+ Müller glia (green) 2 days after NMDA injection (**B**), Smad2/3 activation was observed in Hes5-GFP- cells in the INL (**C**). Scale bars: 30 µm. **D.** NMDA damage activated Smad1/5/8 in remaining retinal ganglion cells and displaced amacrine cells located in the GCL. Scale bars: 10 µm. **E.** Real-time qPCR data showing that NMDA damage induced significant increase in *Bmp4* expression in the retina 2 days after NMDA damage. Expression of other ligands of BMP signaling, *Bmp2* and *−7*, was also induced. *p<0.05 with paired t-test (n = 4). Images shown in **A–D** are representative of at least 3 animals per each treatment group.

Previous studies have shown that growth factors, such as CNTF, LIF, FGF2, and BDNF are upregulated in the retina after damage [1], [2], [3], [4], [5]. However, there has been little characterization of BMP signaling after retinal damage. We therefore examined whether BMP signaling increases following NMDA-induced retinal damage, using antibodies against activated forms of Smad, key downstream components of the BMP and TGF-beta signaling pathways. As shown in Figure 2A, pSmad1/5/8 is barely detectable in the undamaged retina, but there is a substantial increase in the labeling throughout the inner retina at 1 and 2 days after NMDA treatment. Many of the cells in which the BMP signaling has been activated are Müller glia (Figure 2B), as shown by their co-expression of pSmad 1/5/8 and Hes5-GFP [24]. The majority of cells in the ganglion cell layer are also labeled with the pSmad1/5/8 antibody, and these were either displaced amacrine cells or surviving ganglion cells (Figure 2D). The activation of Smad1/5/8 was likely to be mediated by an increased expression of BMP ligands, BMP2, −4, and −7 in the retina following NMDA damage (Figure 2E). *Bmp4* mRNA level was increased by 2.07±0.33 fold 2 days after NMDA damage compared to untreated retinas. Smad2/3 is also activated after damage, indicating that TGF-beta signaling is also increased after damage, though fewer cells are labeled with pSmad2/3 than for pSmad1/5/8. While most of the pSmad2/3 labeled cells in the INL, Hes5-GFP+ Müller glia were not labeled with the pSmad2/3 antibody (Figure 2C).

**Figure 3:**
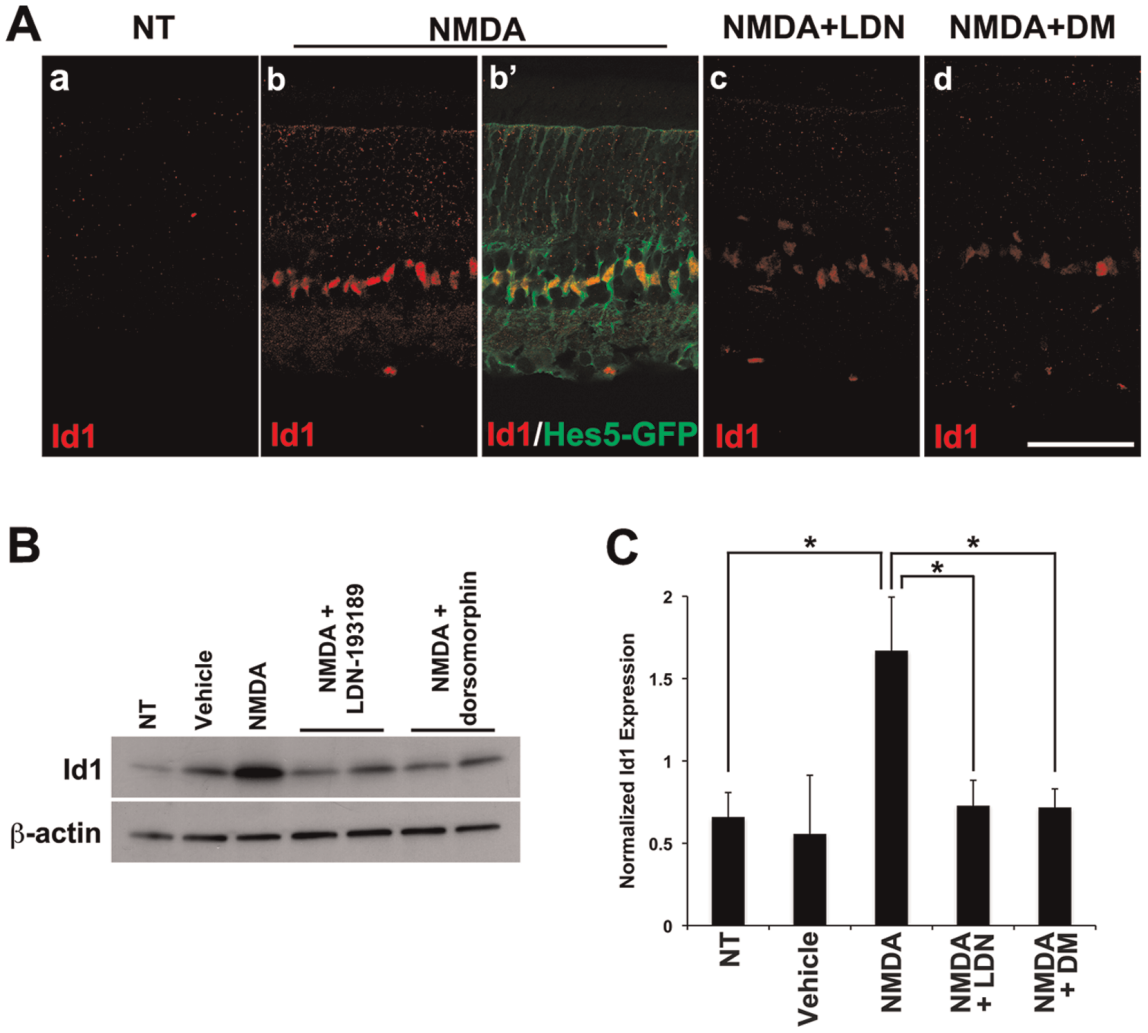
NMDA damage induces expression of Id1, a known target of BMP-Smad1/5/8 signaling. **A.** Representative images from at least 3 animals per treatment are shown. Injection of 100 mM NMDA induced Id1 expression (red) in Hes5-GFP+ Müller cells (green). Id1 expression was blocked by coinjection of BMP inhibitors, LDN-193189 (LDN) or dorsomorphin (DM). Scale bar: 50 µm. **B.** Representative Western blot showing the level of Id1 expression 2 days after injection of the indicated factors. **C.** Quantification of the Western blots. More than a 2 fold increase in Id1 expression was observed after NMDA damage, and this increase was blocked effectively by LDN or DM. The level of Id1 expression was normalized to beta-actin. *p<0.05 (t-test; n = 3 retinas).

To verify that the increase in BMP signaling we observe with the pSmad1/5/8 antibody is able to activate known targets of this pathway, we assayed Id1, a well-characterized downstream target of BMP/Smad1/5/8 (Figure 3) [28], [29]. Id1 is not detectable in the undamaged retina, but NMDA damage induces robust labeling of Id1 two days after the NMDA treatment. As for the pSmad1/5/8, the Id1 expression is present in the nuclei of Hes5-GFP+ Müller glia (Figure 3A). The addition of either of two different well-characterized BMP receptor inhibitors, LDN-193189 (LDN) or dorsomorphin (DM), substantially reduced the NMDA-induced expression of Id1 in Müller glia. Western blot analysis supported the results we observed in retinal sections (Figure 3B–C). NMDA treatment caused a significant increase in the level of Id1 in the retina (approximately 2.5 fold increase compared to NT) (Figure 3C) and this was almost completely blocked by co-injection of either LDN-193189 or DM.

**Figure 4:**
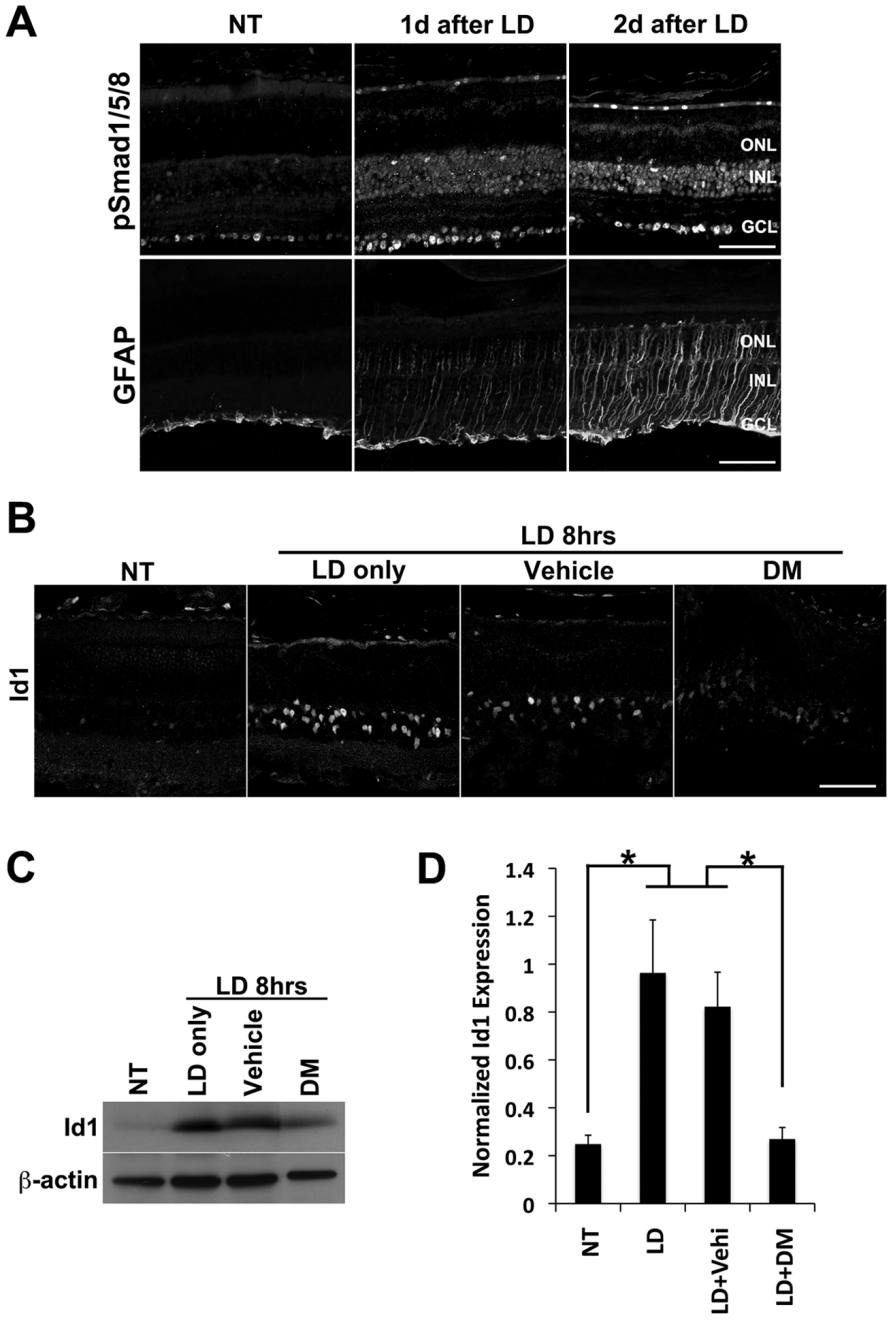
BMP-Smad1/5/8 signaling is activated in the retina after light damage. **A.** Exposure to bright light (light damage; LD) is known to induce a significant loss of photoreceptor cells in the ONL. Two days after 8 hrs of LD, there was strong increase in GFAP expression, suggesting the retina was under stress. Smad1/5/8 activation (pSmad 1/5/8) was also observed in retinal ganglion cells and cells in the INL. ONL, outer nuclear layer; INL, inner nuclear layer; GCL, ganclion cell layer. Scale bars: 50 µm. **B.** 8 hrs of LD induced Id1 expression and this was blocked by injection of the BMP inhibitor, DM. Scale bar: 50 µm. **C.** Representative Western blot showing the level of Id1 expression after the indicated treatments. **D.** Id1 expression was induced by LD (3.3 fold increase compared to NT), and the expression was effectively blocked by injection of DM along with LD. The level of Id1 expression was normalized to beta-actin. Y axis shows an arbitrary value. *p<0.05 (t-test; n =  at least 3 retinas per treatment). Images shown in **A** and **B** are representative of at least 3 animals per each treatment group.

To determine whether the activation of BMP signaling in the inner retina after NMDA damage is specific to this type of retinal injury, or alternatively is a more general response, we also carried out a series of light damage experiments. Albino mice were exposed to intense light for 8 hrs to induce rod photoreceptor loss. As shown in Figure 4A, light damage caused an increase in pSmad1/5/8 at both 1 and 2 days after exposure. The increase in the number and intensity of pSmad1/5/8 labeled cells paralleled the level of GFAP expression in the Müller glia. As in the NMDA damaged retinas, the pSmad1/5/8 labeled cells were found in both the inner nuclear layer and the ganglion cell layer. There was also a 3.3-fold increase in Id1 expression in the Müller glia after light damage, and this effect could be blocked by a co-injection of DM (Figure 4B–D). The effects of light damage on Id1 expression and its inhibition by DM were observed in sections (Figure 4B) and by Western blot (Figure 4C–D).

### Inhibitors of the BMP receptor effectively block NMDA-induced phosphorylation of Smad1/5/8

**Figure 5:**
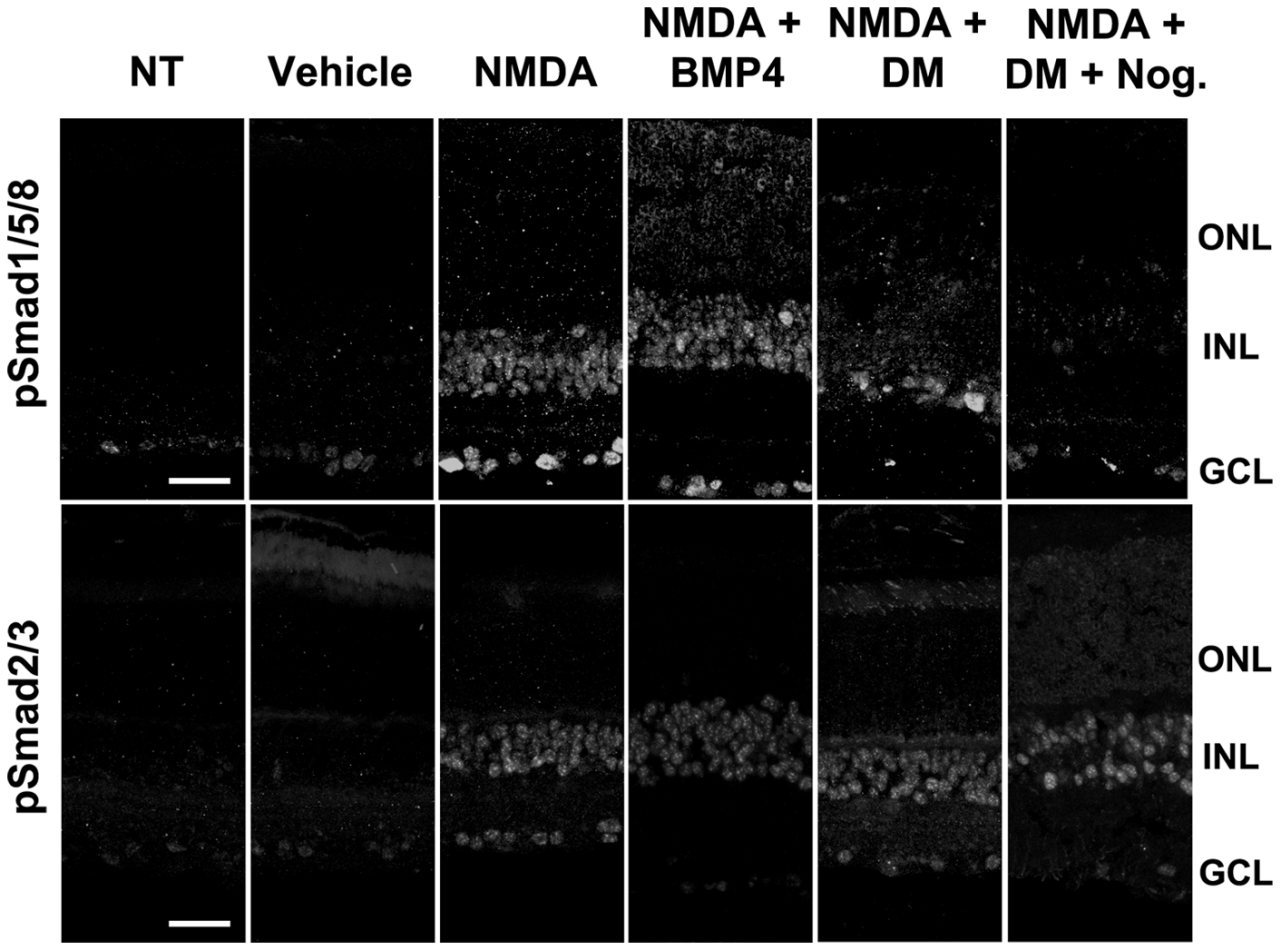
Injection of BMP4 or inhibitors of BMP along with NMDA potentiates or decreases, respectively, Smad1/5/8 activation without affecting Smad2/3 signaling. Smad1/5/8 and Smad2/3 activation was detected 2 days after the injection of indicated factors by immunohistochemistry. A lower dose of NMDA (10 mM) sufficiently activates both Smad1/5/8 and Smad2/3 in the inner retina. Injection of BMP4 along with NMDA caused a small increase in pSmad1/5/8 over the NMDA alone. When NMDA was co-injected with DM, Smad1/5/8 phosphorylation was blocked effectively without affecting Smad2/3 activation. Co-injection with an additional BMP signaling inhibitor, noggin (Nog), completely blocked Smad1/5/8 activation, and caused slight reduction in pSmad2/3. ONL, outer nuclear layer; INL, inner nuclear layer; GCL, ganclion cell layer. Scale bars: 30 µm. Representative images from at least 3 animals per treatment are shown.

To assess the specificity of the BMP receptor inhibitors, we analyzed sections of retinas for pSmad1/5/8 and pSmad2/3 after intravitreal injections of NMDA alone, BMP4 co-injection with NMDA, or NMDA treatment with co-injection of the BMP receptor blocker, DM and the natural BMP inhibitor, noggin (Figure 5). In untreated retinas, there is typically a low level of pSmad1/5/8 labeling in the ganglion cell layer (Figure 5), but cells in the inner nuclear layer are not labeled. Either a low dose (10 mM; Figure 5) or a high dose (100 mM; Figure 2A) of NMDA treatment induced robust labeling of both pSmad1/5/8 and pSmad2/3 after 2 days, particularly in cells of the inner nuclear layer (Figure 5, arrows). Co-injection with BMP4 caused a small, but reproducible increase in the pSmad1/5/8 labeled cells, but no change in pSmad2/3. Co-injection of NMDA with either DM or the combination of DM and noggin, led to a marked inhibition in the number of pSmad1/5/8 labeled cells in the INL, but this treatment had only a slight effect on the pSmad 2/3 labeling. These data confirm the specificity of the BMP inhibitors for this pathway, and also demonstrate that the combination of DM and noggin is most effective for complete inhibition of BMP signaling after NMDA damage.

### Activation of BMP-Smad1/5/8 signaling promotes survival of retinal ganglion cells in NMDA damage

**Figure 6:**
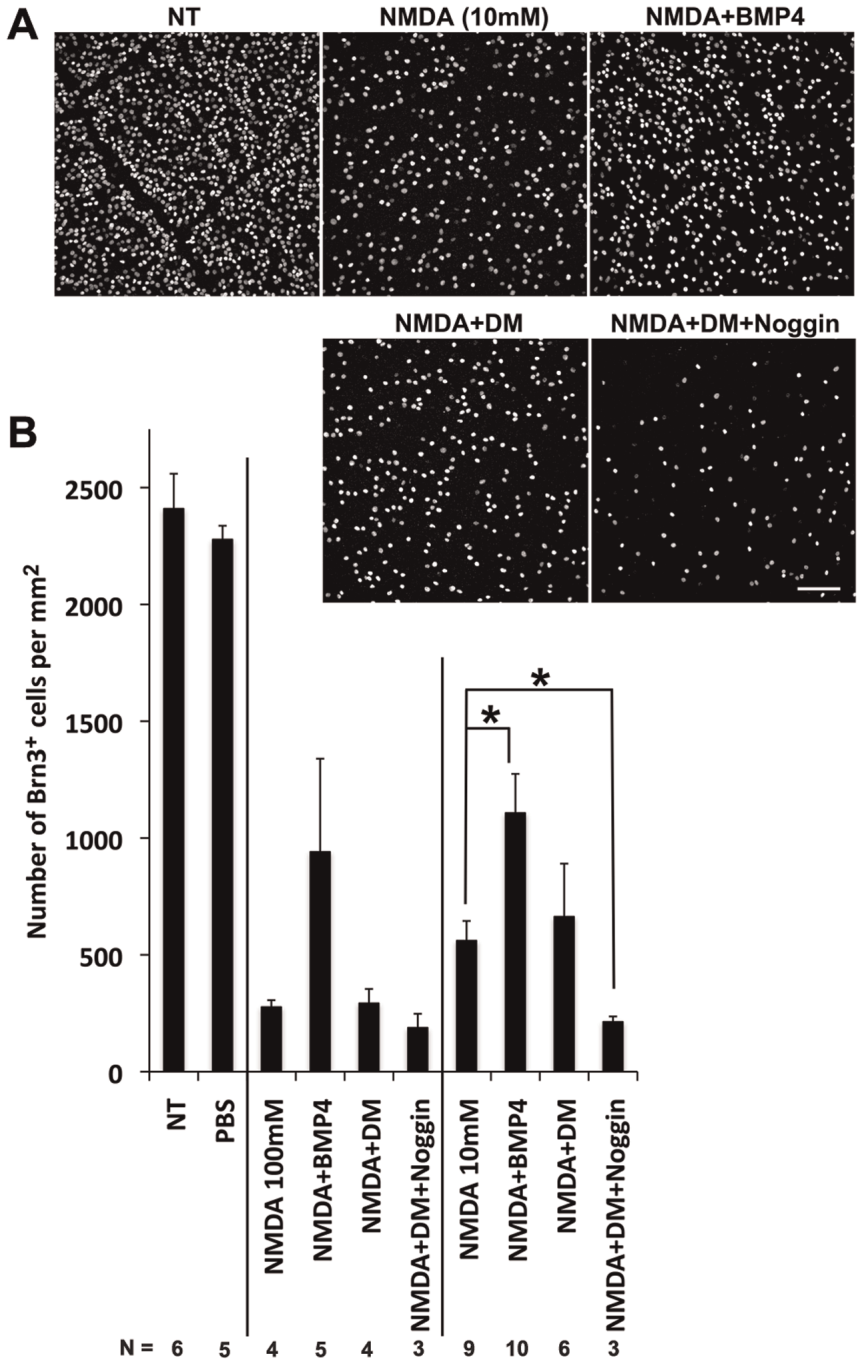
BMP-Smad1/5/8 signaling promotes survival of retinal ganglion cells *in vivo* after NMDA damage. **A.** Retinas were collected 2 days after NMDA injection (10 mM or 100 mM) with or without indicated factors, and then immunostained for Brn3, a marker for retinal ganglion cells. Representative images of retinal flatmouts are shown. Scale bar: 100 µm. **B.** Brn3+ cells were counted in random flatmount fields. In 10 mM NMDA damage, there were significantly more Brn3+ cells when BMP4 was co-injected with NMDA. On the other hand, co-injection of BMP inhibitors (NMDA+DM+Noggin) showed a significant reduction in Brn3+ cells compared to NMDA injection alone. The same trend was observed for the more severe NMDA damage (100 mM). *p<0.01 (t-test). Number of retinas analyzed for each treatment is shown in **B**.

The increase in BMP signaling in the inner retina after retinal injury is similar to previously reported changes in other neuroprotective growth factors, such as CNTF [2], [3], [4], [5]. Therefore, we assessed whether BMP could also act as a neuroprotectant in the retina. We tested this possibility for BMP after NMDA induced ganglion cell death (Figure 6). Mice received intravitreal injections of mild or high doses of NMDA: 10 mM or 100 mM. Some animals also received co-injections of BMP4, DM, or the combination of DM and noggin. As noted above, intravitreal injection of 100 mM NMDA caused the death of almost 90% of the Brn3+ ganglion cells (276.5±29.6 Brn3+ cells/mm^2^ for 100 mM NMDA and 2409.5±149.9 Brn3+ cells/mm^2^ for untreated control) (Figure 6B). Although 10 mM NMDA injection resulted in less damage, approximately 80% of the Brn3+ cells were lost with the lower dose (560.8±84.3 Brn3+ cells/mm^2^ for 10 mM NMDA), Nevertheless, co-injection of BMP4 with either concentration of NMDA protected ganglion cells (Figure 6 A–B). In the higher dose of NMDA, the protection afforded by the BMP4 was more variable (940.8±398.9 Brn3+ cells/mm^2^), and did not reach statistical significance; however, the neuroprotective effect of BMP4 was statistically significant in the 10 mM treatment group (1107.0±167.7 Brn3+ cells/mm^2^) (Figure 6B).

The above results show that BMP4 can act as a neuroprotectant for ganglion cells that would otherwise die from NMDA neurotoxicity. To determine whether the endogenous BMP-Smad1/5/8 signaling that occurs following NMDA injury (Figure 2, 3, 5) provides this same benefit, we co-injected BMP inhibitors with NMDA and assessed the effects on Brn3+ ganglion cell survival. We found that blocking endogenous BMP signaling with the combination of DM and noggin led to a further decline in the number of surviving ganglion cells after 10 mM NMDA treatment (Figure 6 A–B). The number of surviving ganglion cells in the DM/noggin treated group with 10 mM NMDA (213.0±23.1 Brn3+ cells/mm^2^) was similar to that observed after treatment with 100 mM NMDA (188.0±59.9 Brn3+ cells/mm^2^). In the 100 mM NMDA treatment group, 10% of the Brn3+ ganglion cells were resistant to even high doses of NMDA, and inhibition of BMP signaling has no effect on these cells. Taken together, our results suggest that the endogenous BMP signaling that occurs following retinal injury serves a neuroprotectant function.

## Discussion

The results of our analysis show that retinal injury, either by NMDA treatment or by exposure to bright light, causes an increase in BMP expression and BMP signaling in inner retinal cells. The phosphorylation of Smad1/5/8, a key downstream component of the BMP signaling pathway, occurs in Müller glia and inner retinal neurons in response to light damage or NMDA treatment. This suggests that BMP signaling is a general response to retinal injury. The results of experiments with BMP inhibitors indicate that activation of BMP-Smad1/5/8 signaling after damage provides some neuroprotective benefit to retinal ganglion cells.

### BMP signaling regulates many aspects of the eye development, including patterning, growth and apoptosis

It has been shown that BMP2, −4 and −7 and their receptors, BMPRs, are expressed and play essential roles during eye development [30], [31]. For example, BMP4 deficient mice (*Bmp4*+/− or −/−) exhibit abnormal optic nerve development and an elevated intraocular pressure, as well as failure in lens induction and anterior segment dysgenesis [18], [32]. On the other hand, excessive BMP4 signaling leads to reduced retinal volume and alteration of the shape of the eye [33]. The role of BMPs in mature retina has not been extensively studied. Expression of BMPs rapidly decreases in postnatal development and becomes undetectable in adult retina [30]. However, there is detectable expression of Bmpr1b in adult retina [17], [30]. Moreover, both *BmprIb* and *BmprII* mRNAs are expressed in the inner nuclear and ganglion cell layers at least as late as postnatal day 10 in mouse retina [19], suggesting that adult retinal cells can activate BMP signaling when ligands are available. Our data supports this since retinal damage or injection of BMP4 can readily activate Smad signaling (Figures 2A and 5).

Previous studies have established a role for BMP signaling in regulating apoptosis during retinal development: in cultured chick embryonic eyes or retinas, exogenous application of BMP4 or −7 promoted apoptosis [17], [22]; this pro-apoptotic effect of BMPs was significantly reduced by treatment with BMP inhibitor, noggin [22]. Treatment of postnatal day 2 mouse retinal whole mounts with BMP4 also induced increased apoptosis, especially in retinal ganglion cell layer [17]. By contrast, an anti-apoptotic role of BMP signaling in developing mouse retina has been shown *in vivo*. *Bmpr1b*−/− mice display increased apoptosis in the inner retina at postnatal day 7 [19]. In addition, conditional ablation of *Smad4*, required for canonical BMP signaling, resulted in increased apoptosis in the embryonic retina [20]. In posthatch chicks, intraocular BMP injections also dramatically reduced the number of apoptotic cells induced by NMDA injections [34]. In this report, we demonstrate that activation of BMP signaling during neurotoxic damage in adult mouse retina promotes survival of retinal ganglion cells *in vivo* (Figure 6). The downstream mechanisms by which BMP regulates the apopotic pathway are likely to be complex, since it can have both positive and negative effects on cell survival.

### Activation of BMP-Smad1/5/8 signaling may be neuroprotective after adult CNS injury

In other regions of adult CNS, BMPs are known to be upregulated following injury. Rapid increases in BMP4 and −7 expression and phosphorylation of Smad1/5/8 were observed at the site of demyelinating lesions in rat spinal cord, which may stimulate glial scar formation [13]. BMP7 expression was increased dramatically in both glial cells and motor neurons in a different spinal cord injury model [14]. In addition, an increase in BMPRII expression was observed in the rat hippocampus after ischemia [35]. It has been shown that treatment with exogenous BMP7 prior to induction of ischemia-induced injury or focal stroke leads to reduction in cerebral infraction and promotes functional recovery [36], [37]. Neuroprotective effects of BMP7 treatment in spinal cord injury have also been demonstrated [38]. These studies suggest a role for BMPs in promoting cell survival in the adult CNS. In this study, we demonstrated that activation of BMP signaling following retinal damage promotes retinal ganglion cell survival *in vivo*, further supporting a neuroprotective role for BMP signaling in the CNS.

### Potential mechanisms of BMP-mediated protection of retinal ganglion cell after damage

It is currently unclear whether the neuroprotective effect of BMP signaling on retinal ganglion cells is due to a direct effect or mediated via Müller glial activation. Treatment of the retina with BMP inhibitors, DM and noggin, along with 10 mM NMDA damage sufficiently blocked Smad1/5/8 activation in both cell types (Figure 5), while increasing sensitivity of retinal ganglion cells to neurotoxic damage (Figure 6).

It has been suggested that BMP signaling plays a role in reactive gliosis that forms the glial scar after spinal cord injuries in adult animals. Expression of BMP2, −4 and −7 is induced and Smad1/5/8 is activated after spinal cord injury [13], [14], [39]. This activation of BMP signaling leads to reactive gliosis and scar formation, possibly through the complex regulation of GFAP expression [39], [40]. Conditional deletions of *Bmpr1a* and *Bmpr1b* in astrocytes show that these receptors have opposite effects on the formation of glial scar: loss of *Bmpr1a* leads to a reduction in glial hypertrophy, whereas loss of *Bmpr1b* leads to hyper-reactive astrocytes [40]. Soon after retinal damage, Müller glia upregulate GFAP [41] and release neurotrophic factors to prevent further tissue damage [42], [43], [44]. In our NMDA and light damage models, strong GFAP expression was induced (Figures 1 and 4A) when BMP-Smad1/5/8 signaling is most active (Figures 2A and 4A). Moreover, we observed that damage induced expression of Id1, a known BMP-Smad1/5/8 target (Figures 3A and 4B). BMP-Smad1/5/8 signaling may have a role in regulating Müller cell reactivity and release of neurotrophic factors, which in turn indirectly promotes ganglion cell survival in retinal damage. This hypothesis is consistent with a previous report in posthatch chick, which found that intraocular injection of BMP prior to NMDA treatment prevented the proliferative Muller glial response that occurs following NMDA damage [34].

It is also possible that BMP interacts with other signaling factors to promote ganglion cell survival. The neuroprotective effects of the members of IL-6 family of cytokines, such as CNTF and LIF, are well characterized [45], [46], [47], [48], [49]. Treatment with CNTF promotes survival of injured retinal ganglion cell *in vivo* [50], [51], [52], [53], [54]. There is evidence that in neural progenitors, LIF and BMP2 act synergistically to induce astrocyte differentiation through forming a complex between STAT3 and Smad1 [55]. A study by Fukuda *et* *al* has shown that LIF stimulates BMP2 expression in STAT3-dependent manner, which further induced Smad1 activation in neuroepithelial cells [56]. A similar mechanism might exist in the mature retina, since injury to the retina leads to both CNTF/LIF upregulation and glial reactivity. Further studies will determine the mechanisms of BMP-induced neuroprotection.

### BMP-Smad1/5/8 signaling may serve as a potential target for neuroprotective therapies

There is a great demand for the development of neuroprotective therapies for retinal degenerations caused by ganglion cell death, such as glaucoma, given that currently available treatments are inadequate. As we demonstrated here, BMP agonists can be potential neuroprotective agents that delay or prevent ganglion cell death in pathogenic conditions. Several neurotrophic factors have been implicated in retinal ganglion cell damage and their survival; intravitreal injection of CNTF, BDNF, or FGF2 all show some degree of ganglion cell protection after ischemic injury in rat retina [10]. Retinal ganglion cell axotomy induces BDNF expression in the frog retina, and its expression further increases with FGF2 treatment [57]. This FGF2-induced BDNF upregulation is important for the long-term survival of retinal ganglion cells [58]. Combining BMPs and these other known protective factors may be useful in enhancing their survival effects on retinal ganglion cells, as they potentially activate several different neuroprotective pathways. For example, the protective effects of neurotrophins (NGF, BFNF, NT3 and −4) are enhanced by co-treatment with BMP6 or −7 in stressed septal cholinergic neurons *in vitro* [59]. Similar protective effects may be obtained by treatment of damaged retinal ganglion cells with BMPs in combination with other neuroprotective factors.
